# Impact of changes in specificity of data recording on cause-specific injury mortality in the United States, 1999–2010

**DOI:** 10.1186/1471-2458-14-1010

**Published:** 2014-09-27

**Authors:** Guoqing Hu, Keita Mamady

**Affiliations:** Department of Epidemiology and Health Statistics, School of Public Health, Central South University, Changsha, China

## Abstract

**Background:**

We aimed to examine changes in the specificity of data recording and assess the impact on cause-specific injury mortality during 1999–2010.

**Methods:**

A longitudinal study was designed to analyze injury mortality data of 1999-2010. Mortality rates for unspecified injury and for cause-specific injury were obtained using CDC’s Web-based Injury Statistics Query and Reporting System (WISQARS™). The proportion of unspecified injury was used to measure the specificity of injury data recording. We used the proportionate method to adjust data specificity and compared changes in cause-specific mortality before and after adjustment.

**Results:**

Between 1999 and 2010, the age-adjusted mortality from unspecified injuries decreased from 3.4 to 2.5 per 100,000 persons for all ages. The proportion of unspecified unintentional injury decreased from 18.9% to 10.9% for the elderly ages 65+. The proportion of unspecified homicide significantly increased for all age groups except ages 25–44 years. After adjustment, increases in age-adjusted mortality rates from falls, poisoning and drowning were less (77 vs. 61%, 66 vs. 51%, and 9 vs. 0%) and decreases in injuries from motor vehicle crashes, suffocation, fire/burn, and natural/environmental disasters were greater (-30 vs. -37%, -17 vs. -24%, -23 vs. -24%, and -46 vs. -51%), respectively. The adjustment resulted in reversed changes in homicide by firearm (-1 vs. 5%) and cut/pierce (-5 vs. 2%), greater increases in homicide by suffocation (9 vs. 16%) for ages 45–64 years, and smaller decreases in all other age- and cause-specific homicide groups.

**Conclusions:**

During 1999–2010, the specificity of data recording changed significantly for homicide rates and elderly unintentional injury mortality and the changes altered trends in cause-specific injury mortality.

## Background

Accurate cause-specific injury statistics are essential for defining the severity of the injury problem, identifying and characterizing influencing factors, and developing effective prevention interventions. When detailed medical documents are lacking, cause-specific injury deaths are often recorded by certifiers as “unspecified injuries” [1]. Of the framework for injury mortality data recommended by U.S. CDC, the category “unspecified number of deaths” is defined as injury cases whose mechanisms are not reported on the death certificate [2]. Many deaths coded as ‘unspecified injury’ were found to come from “fracture, cause unspecified”, “unspecified accidents”, “late effects of unspecified accidents” and “assault by unspecified means” [2]. A study in 2012 reported that current fall mortality data for old adults do not appear to identify all instances of falls because of state variations in coding practice [3].

Two recent studies revealed that the latest large increase in fall mortality among persons age 65 years and older was possibly due to improved data reporting [4, 5], strongly indicating that the quality of data recording has changed; this change may affect all cause-specific injury statistics and further influence the evaluation of cause-specific injury interventions. Unfortunately, these two important issues have not been examined so far.

Data quality of public health surveillance systems reflects the completeness and validity of the data recorded in the surveillance system [6]. Indicators including data completeness, sensitivity, specificity, representativeness, positive predictive value, and positive likelihood ratio are often used to reflect the quality of surveillance system data [6–8]. A full assessment of the completeness and validity of surveillance system data often requires collection of external information on surveillance data through a special study (e.g., a review of sample data, special record linkage, or patient interview) [6].

Due to lack of external information on injury deaths (e.g., verbal autopsy data), we cannot systematically examine the quality of injury mortality data. In this case, as Lu et al. described [1], we used the proportion of unspecified injury deaths as a surrogate indicator to approximately reflect the specificity of data recording, which measured the level of sufficiency and specificity of information for injury causes.

Considering that the recording quality of injury deaths on death certificates was reported to vary with age group and injury intent [9], we proposed two research assumptions: 1) the specificity of injury mortality data changed significantly during 1999–2010 and the changes varied with age group and injury intent; and 2) the changing specificity of mortality data between 1999 and 2010 affected cause-specific injury mortality rates and trends in mortality.

We analyzed mortality rates from 1999 to 2010 to examine changes in the specificity of recorded data and assess the impact on trends in cause-specific injury mortality.

## Methods

### Data source

We obtained mortality rates for unspecified injury and for cause-specific injury using CDC’s Web-based Injury Statistics Query and Reporting System (WISQARS™). WISQARS™ defines “unspecified injuries” using ICD-10 codes: X59 (unintentional); Y09,*U01.9 (homicide); Y35.7 (legal intervention); X84,*U03.9 (suicide); and Y34, Y89.9 (undetermined intent) [10].

### Data analysis

#### a) Changes in specificity of injury mortality data

The proportion of unspecified injury deaths was calculated as “unspecified injury mortality divided by all-injury mortality*100%”. Based on published studies [1, 8], we defined the quality of data specificity as ‘improved’ when the proportion decreased; when the proportion increased, we defined the quality as ‘worsened’.

Based on the results of preliminary analysis that revealed similarity in proportions of unspecified injury for some age groups (not shown here), we divided ages into four groups: 0–24 years, 25–44 years, 45–64 years, and 65+. Because unspecified injuries from legal intervention and with undetermined intent accounted for <8% of all unspecified injuries during 1999–2010, we targeted unspecified injuries of three intent-specific categories: unintentional injury, homicide, and suicide.

Linear regression was used to examine the significance of changes in the proportion of unspecified injury deaths by intent and age group from 1999 to 2010.

#### b) Relationship between changes in mortality rates from unspecified injuries and from cause-specific injuries

For the groups that had high proportions of unspecified injuries and experienced significant changes in the proportion of unspecified injuries between 1999 and 2010, we plotted stacked bar charts to demonstrate changes in mortality rates from unspecified injuries and from cause-specific injuries and performed Spearman correlation to measure the relationship between them. Suicide was excluded from Spearman rank correlation analysis because of very low proportions of unspecified suicide during 1999–2010 (<0.7%).

Based on preliminary analysis (not shown here), seven major unintentional injury causes (falls, motor vehicle traffic crashes/MVT, suffocation, fire/burn, natural/environmental disaster, poisoning, and drowning) and four common homicide mechanisms (firearm, cut/pierce, suffocation, and struck by or against) were targeted for Spearman rank correlation analysis.

#### c) Impact of changes in specificity of data recording

Only intent- and age-specific groups showing significant changes in unspecified injury mortality during 1999–2010 in step a) and significant correlations between unspecified injury mortality and specific injury mortality in step b) were chosen for redistribution of unspecified injury mortality. In total, four groups were redistributed, including unintentional injury, 65+; homicide, 0–24 years; homicide, 45–64 years; and homicide, 65 + .

To quantify the impact of changes in specificity of data recording, we first adjusted the proportion of unspecified injuries at different years to the same level. Of 1999–2010, we chose the year having the lowest proportion of unspecified injuries as reference. We assumed that the proportions of unspecified injuries in other years could be lowered to the reference level if the specificity of injury data recording improved.

For the years not having the lowest proportion, we then redistributed the “excess unspecified injuries” to cause-specific injuries using proportionate method. Early studies indicated that the proportionate method was useful for approximately redistributing injuries with unspecific codes to cause-specific injuries [2, 11, 12]. The proportionate method supposes that the “excess unspecified deaths” have similar cause-specific proportions as injuries with cause-specific codes in the same year. We performed the redistribution of “excess unspecified injuries” by age group and intent.

Finally, we compared the percent change in cause-specific mortality between 1999 and 2010 before and after the redistribution. The percent change in mortality was calculated as “(age-adjusted mortality in 2010 – age-adjusted mortality in 1999)/age-adjusted mortality in 1999*100%”.

The data were from CDC’s Web-based Injury Statistics Query and Reporting System (WISQARS™) that does not cover any private information and could be accessed freely at http://webappa.cdc.gov/sasweb/ncipc/mortrate10_us.html. This study was approved by the IRB of the School of Public Health, Central South University (China) to be exempt for review. Data analysis was performed between March 1, 2014 and May 15, 2014.

## Results

### Changes in specificity of injury mortality data

**Figure 1:**
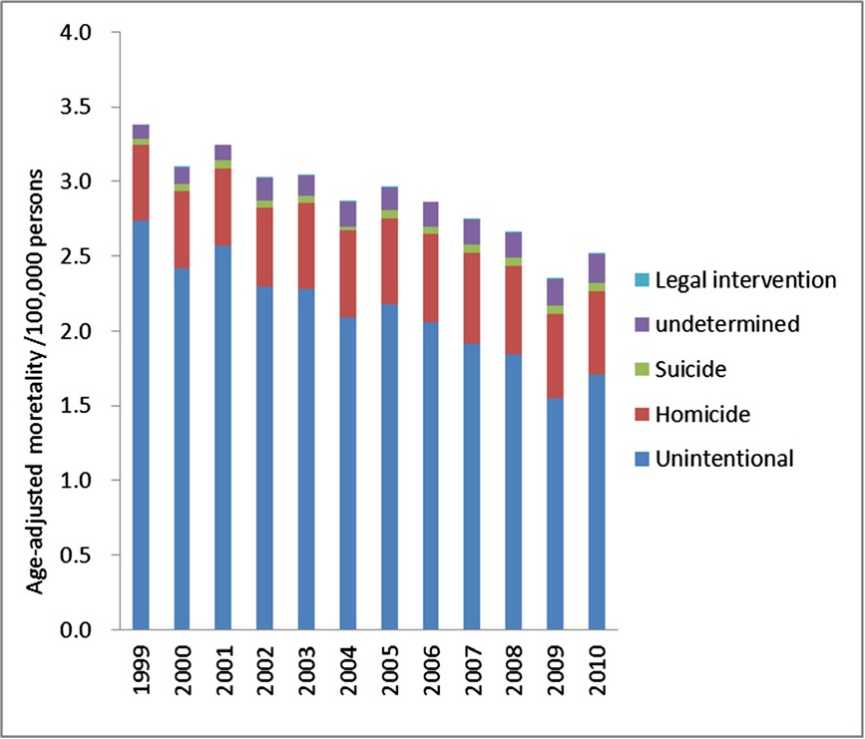
**Age-adjusted mortality /100,000 persons from unspecified injuries by intent (U.S., 1999-2010).**

Between 1999 and 2010, the age-adjusted mortality from unspecified injuries decreased by 26% for all age groups and all intents, changing from 3.4 to 2.5 per 100,000 persons; this led to a drop in the proportion of unspecified injuries from 6% to 4% (Figure 1). Over 92% of unspecified injury deaths were from unintentional injuries and homicide for all years.

**Figure 2:**
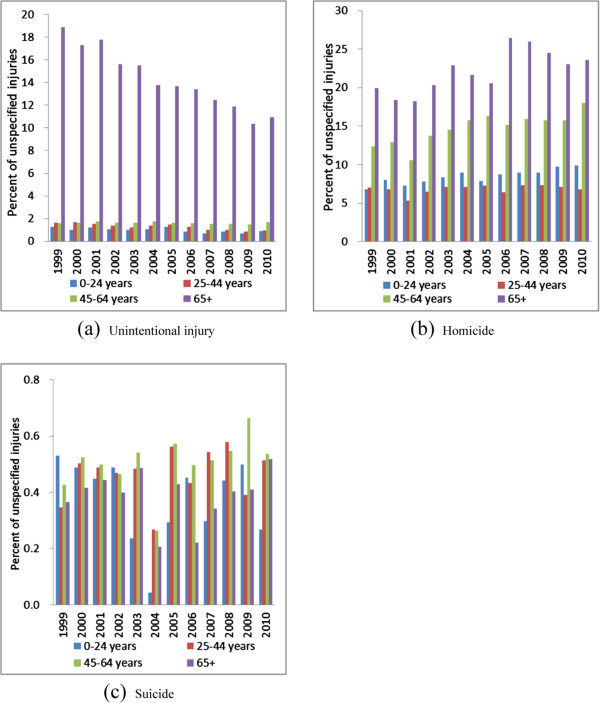
**Percent of unspecified injury mortality among all-injury mortality by intent and age group (United States, 1999-2010). A**. unintentional injury, **B**. homicide, **C**. suicide, 0-24 years.

The age-adjusted mortality from unspecified unintentional injury dropped from 17.7 to 10.9 per 100,000 persons between 1999 and 2010 for the elderly age 65+, causing the proportion to decrease from 18.9% to 10.9%. The proportions for age groups 0–24 years and 25–44 years also decreased but the proportions were under 2% for all years (Figure 2A, Table 1).

**Table 1:** **Linear regression between percent of unspecified injury mortality and year (United States, 1999–2010)**

Intent	Age group	***b***	***s***_***b***_	95% confidence interval		Adjusted ***R*** ^2^
				Lower limit	Upper limit	
Unintentional	0-24 years	-0.037^*^	0.013	-0.066	-0.009	0.413
	25-44 years	-0.077^**^	0.011	-0.101	-0.053	0.816
	45-64 years	-0.006	0.006	-0.020	0.007	0.004
	65+	-0.746^**^	0.047	-0.851	-0.641	0.958
Homicide	0-24 years	0.232^**^	0.034	0.156	0.307	0.807
	25-44 years	0.059	0.046	-0.044	0.163	0.056
	45-64 years	0.483^**^	0.093	0.277	0.690	0.704
	65+	0.578^**^	0.754	0.223	0.932	0.525
Suicide	0-24 years	-0.010	0.012	-0.038	0.018	-0.031
	25-44 years	0.007	0.008	-0.010	0.024	-0.024
	45-64 years	0.011	0.008	-0.006	0.028	0.091
	65+	0.001	0.008	-0.017	0.020	-0.097

^*^: *P* < 0.05; ^**^: *P* < 0.01.

Note: Dependent variable = percent of unspecified injury mortality, independent variable = year.

The proportion of unspecified homicide increased as age increased during 1999–2010. The proportion of unspecified homicide significantly increased for all age groups except ages 25–44 years (Figure 2B, Table 1).

The proportions of unspecified suicide were under 0.7% and fluctuated between 1999 and 2010 for all age groups (Figure 2C, Table 1).

### Relationship between changes in mortality rates from unspecified injuries and from cause-specific injuries

**Figure 3:**
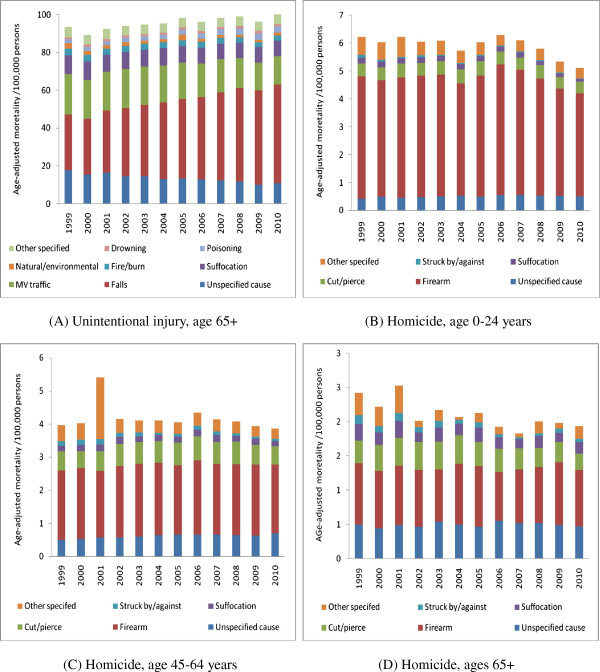
**Age-adjusted injury mortality /100,000 persons by intent and cause (United States, 1999-2010). A**. unintentional injury, age 65+; **B**. homicide, age 0-24 years; **C**. homicide, age 45-64 years; **D**. homicide, ages 65+.

Figure 3A and Spearman correlation revealed that unspecified unintentional injury mortality was negatively correlated with unintentional injuries from falls, poisoning and drowning (*r*_*s*_ = -0.979, -0.989 and -0.796), and was positively correlated with injuries from motor vehicle crashes, suffocation, fire/burn and natural/environmental disasters (*r*_*s*_ = 0.965, 0.916, 0.898 and 0.753) for the elderly age 65+, respectively.

Unspecified homicide was negatively correlated with homicide described as “struck by or against” in age groups 0–24 years and 45–64 years (*r*_*s*_ = -0.713 and -0.643), respectively (Figure 3B-D).

### Impact of changes in specificity of data recording

After adjusting the proportion of unspecified injuries, age-adjusted mortality rates increased more in 1999 than in 2010 for 7 major causes of unintentional injuries among the elderly ages 65+, leading to smaller increases in injuries from falls (77 vs. 61%), poisoning (66 vs. 51%), and drowning (9 vs. 0%) and larger decreases in injuries from motor vehicle crashes (-30 vs. -37%), suffocation (-17 vs. -24%), fire/burn (-23 vs. -24%), and natural/environmental disasters (-46 vs. -51%) (Table 2).

**Table 2:** **Percent change in cause-specific injury mortality/100,000 persons after adjusting unspecified injury (United States, 1999–2010)**

Intent/age group	Cause	Unadjusted			Adjusted		
		1999	2010	% change in rates	1999	2010	% change in rates
Unintentional injury, 65+	Falls	29.4	52.1	77%	32.5	52.4	61%
	MVT	21.5	15.0	-30%	23.8	15.1	-37%
	Suffocation	9.9	8.2	-17%	10.9	8.3	-24%
	Fire/burn	3.5	2.7	-23%	3.7	2.7	-24%
	Natural/environmental	2.8	1.5	-46%	3.1	1.5	-51%
	Poisoning	2.0	3.4	66%	2.3	3.4	51%
	Drowning	1.2	1.3	9%	1.3	1.3	0%
Homicide, 0–24 years	Firearm	4.4	2.7	-16%	4.4	3.8	-13%
	Cut/pierce	0.5	0.4	-11%	0.5	0.4	-8%
	Suffocation	0.2	0.1	-49%	0.2	0.1	-48%
	Struck by or against	0.1	0.02	-83%	0.1	0.02	-82%
Homicide, 45–64 years	Firearm	2.1	2.1	-1%	2.1	2.3	5%
	Cut/pierce	0.59	0.56	-5%	0.60	0.61	2%
	Suffocation	0.15	0.16	9%	0.15	0.18	16%
	Struck by or against	0.15	0.07	-54%	0.15	0.08	-51%
Homicide, 65+	Firearm	0.89	0.82	-8%	0.91	0.88	-3%
	Cut/pierce	0.34	0.24	-28%	0.34	0.26	-25%
	Suffocation	0.24	0.17	-32%	0.25	0.18	-28%
	Struck by or against	0.13	0.05	-61%	0.13	0.05	-59%

Note: % change in rates was calculated as “(age-adjusted mortality in 2010 – age-adjusted mortality in 1999)/age-adjusted mortality in 1999*100%”.

MVT: motor vehicle traffic crashes.

In contrast, the adjustment resulted in more increases in 2010 than in 1999 for cause-specific homicide rates in age groups 0–24 years, 45–64 years and 65+; this resulted in reversed trends in homicide by firearm (-1 vs. 5%) and cut/pierce (-5 vs. 2%) and greater increases in homicide by suffocation (9 vs. 16%) for ages 45–64 years, and smaller decreases in all other age- and cause-specific homicide (Table 2).

## Discussion

As assumed, we found that (1) the specificity of data recording changed distinctly during 1999–2010 and the changes varied with age and intent; (2) changes in specificity of data recording were closely related to changes in mortality rates from some cause-specific unintentional injuries and homicide; and (3) changes in cause specificity affected cause-specific unintentional injury and homicide mortality rates in 1999 and in 2010 and altered trends in cause-specific injury mortality substantially.

These findings have important implications. On the one hand, the results indicate that the specificity of data recording has improved for unintentional injury but worsened for homicide in the last decade. It emphasizes that recent changes in cause-specific injury mortality should be interpreted with caution for age-and intent-specific groups having a high proportion of unspecified injury and experiencing large changes in the specificity of data recording (typically for homicide and elderly unintentional injury). For example, the recently reported increases in injury deaths from falls [13–15] and poisoning [13, 16] were possibly overestimated while the reported decrease in deaths from motor vehicle crashes [13] may be underestimated. The high proportions of unspecified homicide rates among age groups 45–64 years (11-18%) and 65+ (18-27%) also suggest that cause-specific homicide rates may be seriously underestimated for 1999–2010. Encouragingly, the specificity of suicide data has remained at a high level between 1999 and 2010 and unspecified suicide has little impact on cause-specific suicide rates. Variations in specificity between intent-specific groups may reflect differences in coding practice. Dijkhuis et al [9] reported that intentional fatalities were more likely to be investigated by a medical examiner than fatalities from unintentional falls and transportation fatalities among the elderly.

On the other hand, it underscores the value of looking into the quality of injury data recording. Data quality is influenced by the performance of the screening and the case definition for the health-related event, the clarity of hardcopy or electronic surveillance forms, the quality of training and supervision of persons who complete these surveillance forms, and the care exercised in data management [6]. To provide reliable statistics for policy-making, it is necessary to systematically assess the quality of data recording (under-reporting, specificity and misclassification), identify factors influencing the quality of data recording, and develop solutions to improve data quality. At present, it is helpful to quantify the overall impact of changes in data recording on cause-specific injury mortality rates and adjust the reported cause-specific injury mortality rates.

In addition, our findings have potential implications in health resource allocation and utilization, policy evaluation for some other countries and the world. We realize that the quality of mortality statistics has improved in some countries (e.g., China.) but worsened in other countries (e.g., Iraq, Afghanistan and Syria), as the result of socio-economic factors such as economic growth and war. Clearly, changes in data quality (including specificity) can have an effect on health policy-making in these countries, especially for the evaluation of cause-specific health policy changes over time. The potential effect of changes in data quality in these countries further affects the estimation of global mortality statistics, such as the global burden of disease. Unfortunately, the impact of changes in data quality has not been rigorously assessed in countries where data quality improved or worsened. To obtain reliable and valid mortality statistics (not merely injury statistics), it is important for the government of these countries and international organizations such as the World Health organization (WHO) to assess and quantify changes in mortality data quality in the last several decades. Our study is primarily limited by lack of external information on injury deaths. Without the information, we cannot examine the effects of underreporting and misclassification on cause-specific injury mortality. This is critical for generating high-quality injury statistics. Second, we did not assess the impact of specificity changes on injury morbidity. Because of differences in coding practice between mortality data and morbidity data, the impact on injury morbidity data may differ. In addition, we did not analyze changes in specificity within the same injury cause on code-specific injury mortality. Code-specific data provides more details of injury than aggregated cause-specific data. For example, injury from falls includes 20 mechanisms (w00-w19), but the last three categories do not provide the details (w17, other fall from one level to another; w18, other fall on same level; and w19, unspecified fall) [17]. Recent studies reported substantial changes in code-specific fall mortality for the elderly ages 65 + [4, 5].

## Conclusions

We conclude that the specificity of data recording changed significantly for homicide rates and elderly unintentional injury mortality rates during 1999–2010 and the changes yielded an important impact on changes in cause-specific injury mortality. Policy-makers and researchers should interpret recent trends in cause-specific injury mortality with caution. Further studies are needed to quantify and adjust the overall impact of changes in data recording (including under-reporting, specificity and misclassification) on reported cause-specific injury statistics, in order to provide high-quality statistics for policy-making.
